# Diagnostic value of BHI-V4 for heterogeneous and vancomycin-intermediate *Staphylococcus aureus* isolates: a systematic review and meta-analysis

**DOI:** 10.1186/s12879-024-09274-4

**Published:** 2024-05-14

**Authors:** Xin Cheng, Juntong Zhou, Fan Yuan, Jingxin Ma, Shuilong Guo, Jianrong Su

**Affiliations:** 1 Clinical Laboratory Center, Beijing Friendship Hospital, Capital Medical University, Beijing, China; 2 Department of Otolaryngology, Beijing Friendship Hospital, Capital Medical University, Beijing, China; 3 Department of Gastroenterology, Beijing Friendship Hospital, Capital Medical University, Beijing, China

**Keywords:** hVISA, VISA, BHI-V4, Diagnostic value, Systematic review, Meta-analysis

## Abstract

**Background:**

Brain-heart infusion agar supplemented with 4 µg/mL of vancomycin (BHI-V4) was commonly used for the detection of heterogeneous (hVISA) and vancomycin-intermediate *Staphylococcus aureus* (VISA). However, its diagnostic value remains unclear. This study aims to compare the diagnostic accuracy of BHI-V4 with population analysis profiling with area under the curve (PAP-AUC) in hVISA/VISA.

**Methods:**

The protocol of this study was registered in INPLASY (INPLASY2023120069). The PubMed and Cochrane Library databases were searched from inception to October 2023. Review Manager 5.4 was used for data visualization in the quality assessment, and STATA17.0 (MP) was used for statistical analysis.

**Results:**

In total, eight publications including 2153 strains were incorporated into the meta-analysis. Significant heterogeneity was evident although a threshold effect was not detected across the eight studies. The summary receiver operating characteristic (SROC) was 0.77 (95% confidence interval [CI], 0.74–0.81). The pooled sensitivity, specificity, positive likelihood ratio, negative likelihood ratio, diagnostic score and diagnostic odds ratio were 0.59 (95% CI: 0.46–0.71), 0.96 (95%CI: 0.83–0.99), 14.0 (95% CI, 3.4–57.1), 0.43 (95%CI, 0.32–0.57), 3.48(95%CI, 2.12–4.85) and 32.62 (95%CI, 8.31-128.36), respectively.

**Conclusion:**

Our study showed that BHI-V4 had moderate diagnostic accuracy for diagnosing hVISA/VISA. However, more high-quality studies are needed to assess the clinical utility of BHI-V4.

**Supplementary Information:**

The online version contains supplementary material available at 10.1186/s12879-024-09274-4.

## Introduction

*Staphylococcus aureus* presents a significant threat to human health as it is capable of causing a wide range spectrum of infections from minor skin conditions to life-threatening diseases, particularly in healthcare settings. According to a survey conducted in 2019, *Staphylococcus aureus* ranked first in global mortality rates among 33 bacterial pathogens [1]. This bacterium has developed resistance to multiple antibiotics, making it difficult to treat and increasing the risk of severe and prolonged infections. Initially, vancomycin was considered the last resort for treating multidrug-resistant *Staphylococcus aureus* (MRSA) [2]. However, the subsequent discovery of heterogeneous (hVISA) and vancomycin-intermediate Staphylococcus aureus (VISA) has posed new challenges in the clinical treatment of these pathogens [3]. Studies have shown that VISA/hVISA was associated with persistent infection, treatment failure, and prolonged hospital stays [4].

VISA/hVISA and vancomycin-resistant *Staphylococcus aureus* (VRSA) are collectively known as vancomycin-insensitive *Staphylococcus aureus*. In clinical practice, VRSA is rare, while VISA/hVISA is more commonly encountered. However, the main challenge for VISA/hVISA lies in its identification. hVISA consists of subpopulations with a frequency of 10^− 4^-10^− 6^, exhibiting varying levels of vancomycin-intermediate resistance. Standard antimicrobial susceptibility testing methods, such as the broth microdilution method (BMD) and agar dilution method (AD), utilize a two-fold dilution system, which may mistakenly classify hVISA as vancomycin-sensitive *Staphylococcus aureus* (VSSA). In addition, the disk diffusion method is not recommended by the Clinical and Laboratory Standards Institute (CLSI) as a screening method for vancomycin resistance due to the slow diffusion of vancomycin in the culture medium [5]. Different methods commonly used for detecting the minimum inhibitory concentration (MIC) of vancomycin also show variations. Etest results are twofold higher than broth microdilution (BMD) MIC results, while Vitek2 results are twofold lower than BMD MIC results [6]. When using the broth dilution method as the standard, Vitek Legacy and Vitek 2 systems tend to classify VISA strains as susceptible, while MicroScan and Phoenix systems, as well as Etest, tend to classify susceptible strains as VISA [7]. In conclusion, traditional drug sensitivity testing is not able to reliably identify hVISA/VISA.

Furthermore, the “gold standard” for hVISA/ VISA detection is population analysis profiling with area under the curve (PAP-AUC) [8]. However, this method is time-consuming and labor-intensive, making it unsuitable for routine clinical use.

The routine screening for hVISA/VISA is mainly conducted through phenotypic analysis using BHIA with different glycopeptides. Numerous studies made extensive use of BHI-V4 due to its affordability and ease of use. However, the diagnostic value of this screening plate remains unclear. To the best of our knowledge, there are few meta-analyses available regarding the screening methods for hVISA/VISA in English journals. As a result, it becomes necessary to summarize the reported findings, assess the available data, and attempt to identify research gaps that need to be filled.

## Materials and methods

### Search strategy

**Fig. 1:**
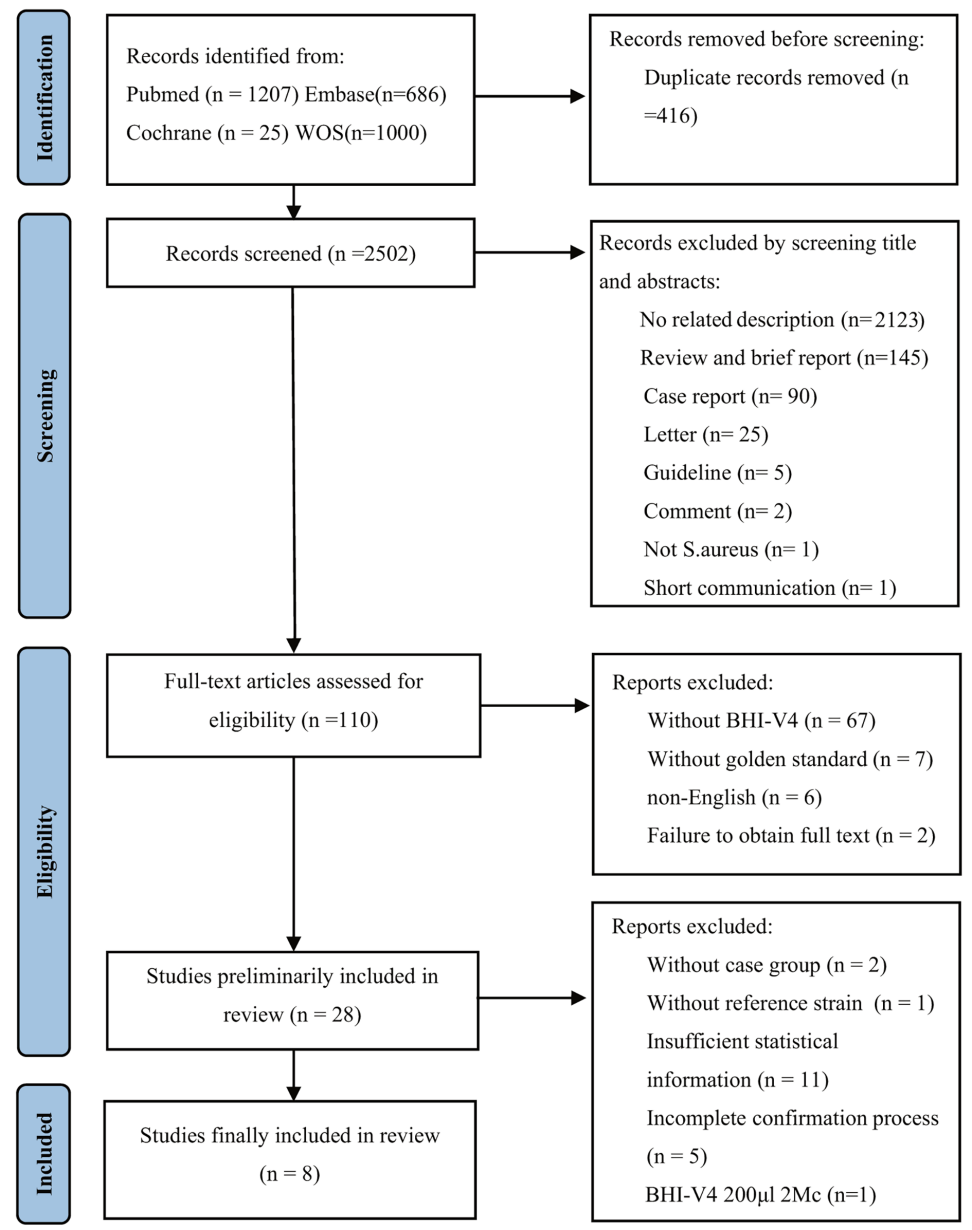
Flow diagram showing the study selection process. WOS: Web of Science

Relevant studies on VISA and hVISA published from inception to October 2023 were searched in PubMed, Embase, Cochrane Library and Web of Science using the following keywords: ‘vancomycin-intermediate *Staphylococcus aureus*’; ‘VISA’; ‘heterogeneous vancomycin-intermediate *Staphylococcus aureus*’; ‘hVISA’; ‘*Staphylococcus aureus* with reduced susceptibilities to vancomycin’; ‘VNSA’; ‘glycopeptide-intermediate *Staphylococcus aureus*’; ‘GISA’; ‘heterogeneous glycopeptide-intermediate *Staphylococcus aureus*’; ‘hGISA’. Figure 1 performs the search strategy outlined following the Preferred Reporting Items for Systematic Reviews and Meta-Analyses (PRISMA) guidelines [9]. The systematic search conducted was not restricted to any specific study or publication type to ensure a thorough evaluation of the literature. Our protocol was registered on 18 December 2023 by INPLASY under registration number INPLASY2023120069 with the purpose of “diagnostic value of BHI-V4 for heterogeneous and vancomycin-intermediate Staphylococcus aureus isolates” [10].

### Inclusion and exclusion criteria

Original studies that met the following criteria were included: (1) all experiments including BHI-V4 screening (The standard inoculum concentration and volume are 0.5McFarland and 10ul, respectively); (2) published papers; (3) reference method was PAP-AUC; (4) The indicators of true positive (TP), false positive (FP), false negative (FN), and true negative (TN) needed for the combined effect value could be derived directly or indirectly using the original study’s data.

Exclusion criteria included: (1) review, brief report, case report, letter, comment, or conference paper; (2) duplicate publications; (3) unable to retrieve full text; (4) non-English publications; (5) unable to obtain the TP, FP, FN, TN, and other data directly or indirectly, or the data is incorrect; (6) without PAP-AUC.

### Study selection

Two reviewers (X.C. and J.Z.) autonomously evaluated titles, abstracts, and full-text papers. Discrepancies in the selection of studies were handled by engaging in discussions until an agreement was reached. If necessary, a third reviewer (F.Y., J.M., S.G., or J.S.) was consulted for further input. The eligibility of studies was evaluated based on the specified criteria for inclusion and exclusion.

### Quality assessment and data extraction

Two reviewers (X.C. and J.Z.) conducted a quality assessment for each study using the QUADAS-2 tool, which is a method for evaluating the quality of diagnostic studies [11]. Review Manager 5.4 was utilized for data visualization in the quality assessment. The following data were extracted from the included studies: author, year, TP, FP, FN, TN, country, sample size, and phenotype, as well as study type.

### Data analysis

STATA17.0 (MP) was utilized for statistical analysis. The effect sizes of diagnostic accuracy were pooled, which included sensitivity, specificity, diagnostic odds ratio (DOR), negative likelihood ratio, and positive likelihood ratio with their associated 95% confidence intervals (95%CIs). The SROC curves were employed to compute the AUC of the integrated model. The threshold heterogeneity of the included studies was assessed using Spearman’s correlation coefficient, and non-threshold heterogeneity was assessed using Cochran’s Q and *I*^*2*^ values. A random effects model was employed to integrate the data if the *I*^*2*^ value was greater than 50% or *P* < 0.05 which indicated significant heterogeneity. When the *I*^*2*^ value was less than 50% or *P* ≥ 0.05, a fixed effect model was chosen. Fagan nomogram was used to evaluate clinical utility. Deek’s funnel plot was used to examine the publication bias of the included studies. A *P* < 0.05 was deemed statistically significant.

## Results

### Search results and study characteristics

The study selection process is depicted in Fig. 1. A total of 1232 studies were yielded by our search approach, with 11 duplicates discarded, 1105 eliminated after titles and abstracts were screened, and 88 removed after full-text article reading. The remaining 28 potentially eligible studies were selected for further evaluation. Of these, 20 articles were excluded based on the exclusion criteria. Finally, a total of eight publications [12–19] were included in this meta-analysis.

The main characteristics of the included studies are briefly presented in Table 1. Of the eight studies, five were retrospective studies and three were prospective studies; half of the studies were conducted in America, whereas the other half were in India, Argentina, Brazil, and England; all studies were conducted during 2001–2017. In addition, the sample size ranged from 11 cases to 500 cases.

**Table 1:** The main characteristics of the included studies

Author	Year	TP	FP	FN	TN	Country	Sample size and phenotype	Study type
Rajesh et al [12]	2017	36	30	26	408	India	500 MRSA	retrospective
Riad et al [13]	2015	37	6	29	544	America	616 MRSA	prospective
Riederer et al [14]	2011	11	0	29	445	America	485 MRSA	prospective
Sabrina et al [15]	2015	0	2	3	6	Argentina	11 unknown	prospective
Sandra et al [16]	2013	15	15	10	20	America	60 MRSA	retrospective
Sarah et al [17]	2010	19	8	2	111	America	140 MRSA	retrospective
Thaina et al [18]	2016	4	0	3	5	Brazil	12 MRSA	retrospective
Timothy et al [19]	2001	32	34	13	250	England	284MRSA 45 SRSV^a^	retrospective

^a^Staphylococcus strains with reduced susceptibilities to vancomycin

### Quality assessment

**Fig. 2:**
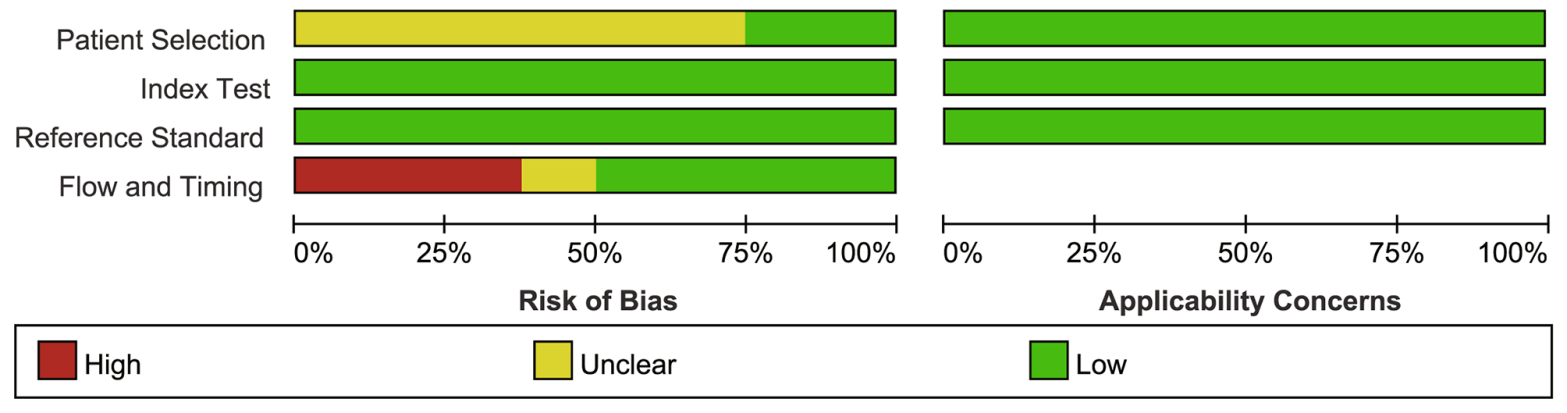
Summary of risk of bias and applicability of concerns graph

As indicated in Fig. 2, the general quality of the included literature was high. The gold standard, diagnostic criteria for hVISA/VISA, and demographic information were all described in all of the included investigations. However, none of the studies adopted a case-control design and it is unclear whether BHI-V4 was conducted at the same time as the reference standard. None of the studies was excluded based on the quality assessment.

### Performance of BHI-V4 in diagnosing hVISA/VISA

#### Heterogeneity analysis

The Spearman’s correlation coefficient was − 0.70, and the proportion of heterogeneity likely due to the threshold effect was 0.48, suggesting that the threshold effect is negligible, thus permitting the combining of the effect sizes. Moreover, the results indicated high non-threshold heterogeneity (*Q* = 99.875, *I*^*2*^ = 98.95%); therefore, the effect sizes were combined using the random effects model.

#### Combined effect analysis

**Fig. 3:**
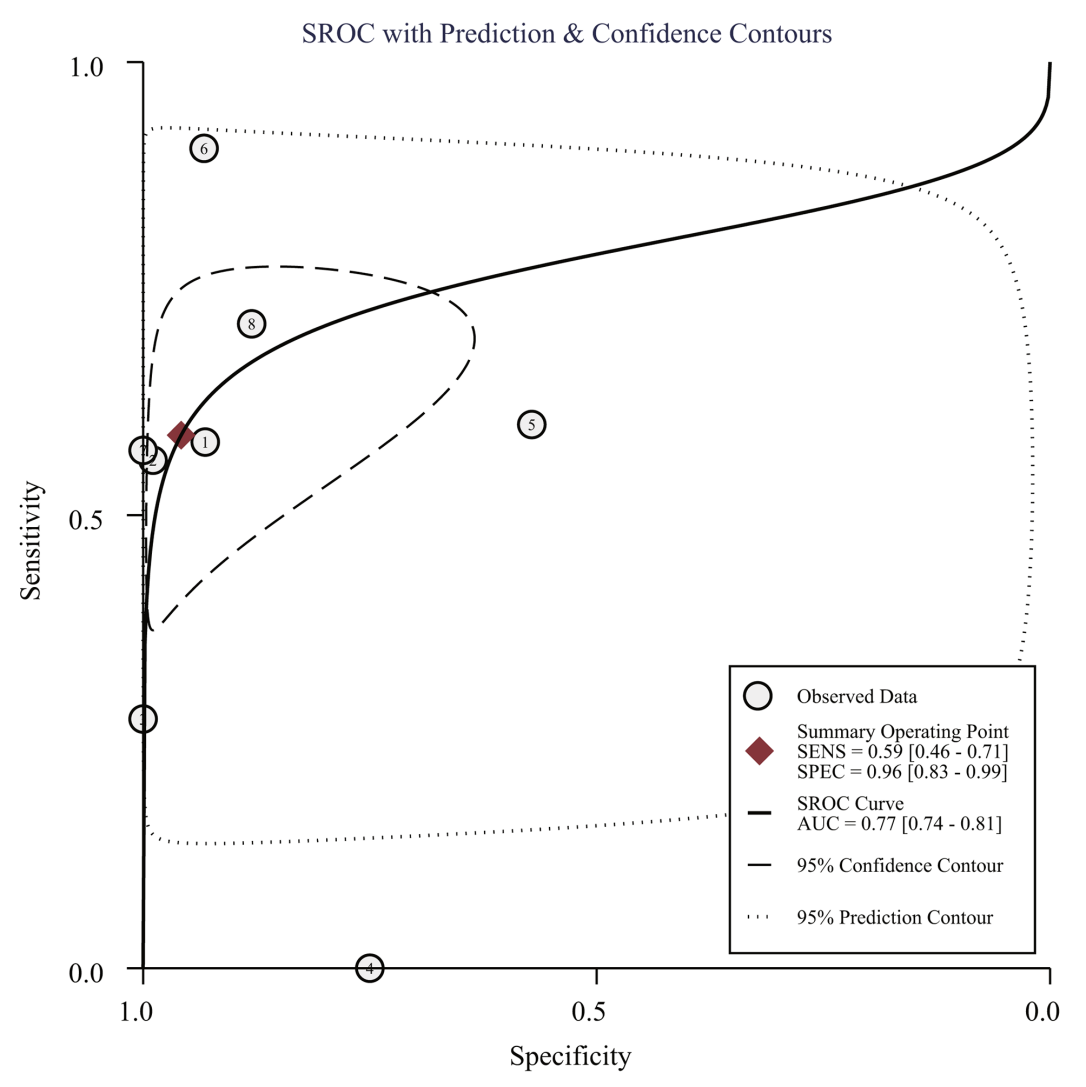
Summary receiver operating characteristic curves

**Fig. 4:**
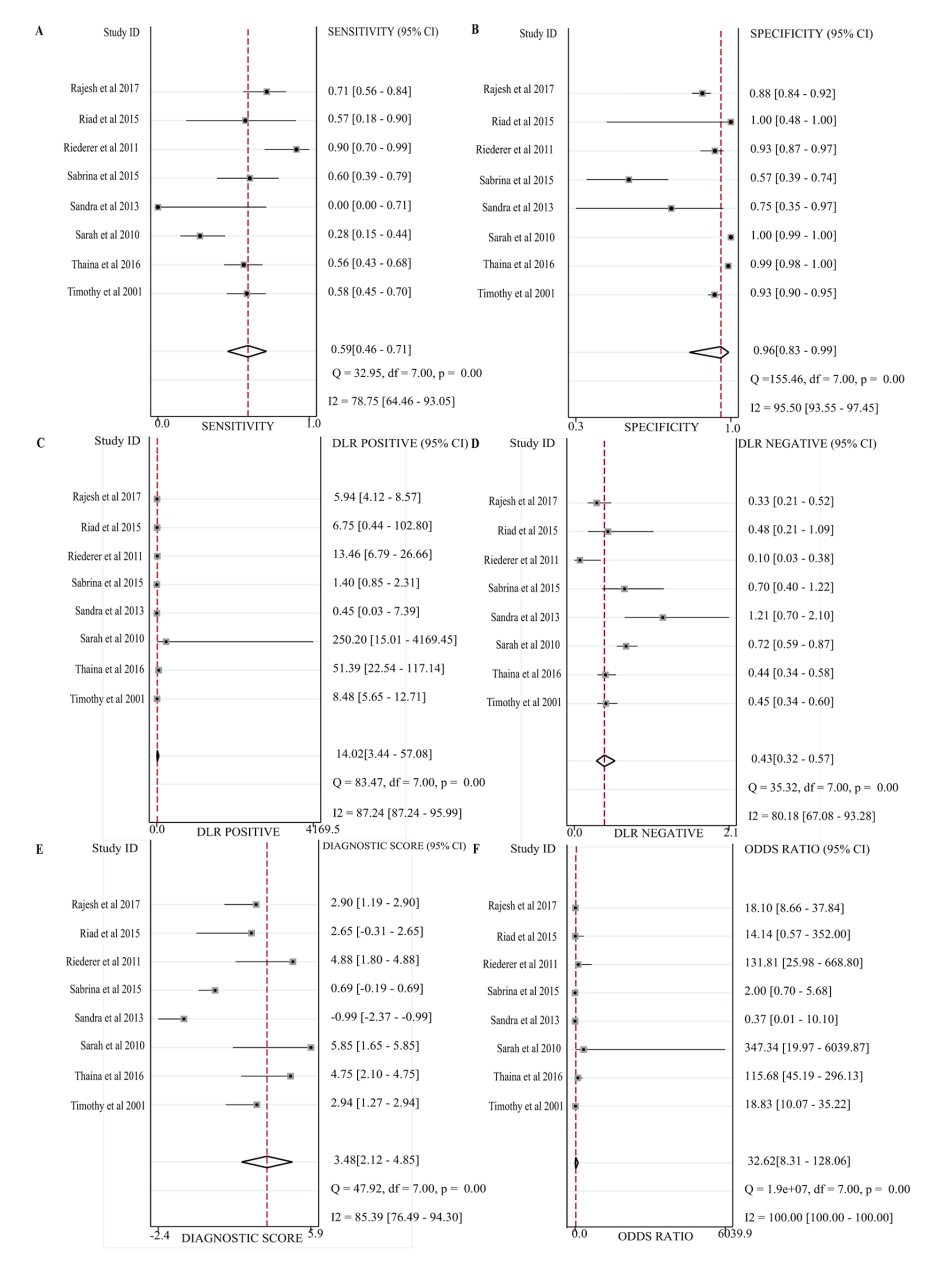
Forest plot of sensitivity **(A)**, specificity **(B)**, positive likelihood ratio **(C)**, negative likelihood ratio **(D)**, diagnostic score **(E)** and diagnostic odds ratio **(F)** of BHI-V4

The SROC was 0.77 (95% CI, 0.74–0.81) (Fig. 3). The pooled sensitivity, specificity, positive likelihood ratio, negative likelihood ratio, diagnostic score, and diagnostic odds ratio were 0.59 (95% CI: 0.46–0.71), 0.96 (95%CI: 0.83–0.99), 14.0 (95% CI, 3.4–57.1), 0.43 (95%CI, 0.32–0.57), 3.48(95%CI, 2.12–4.85) and 32.62 (95%CI, 8.31-128.36), respectively (Fig. 4).

#### Fagan nomogram analysis

**Fig. 5:**
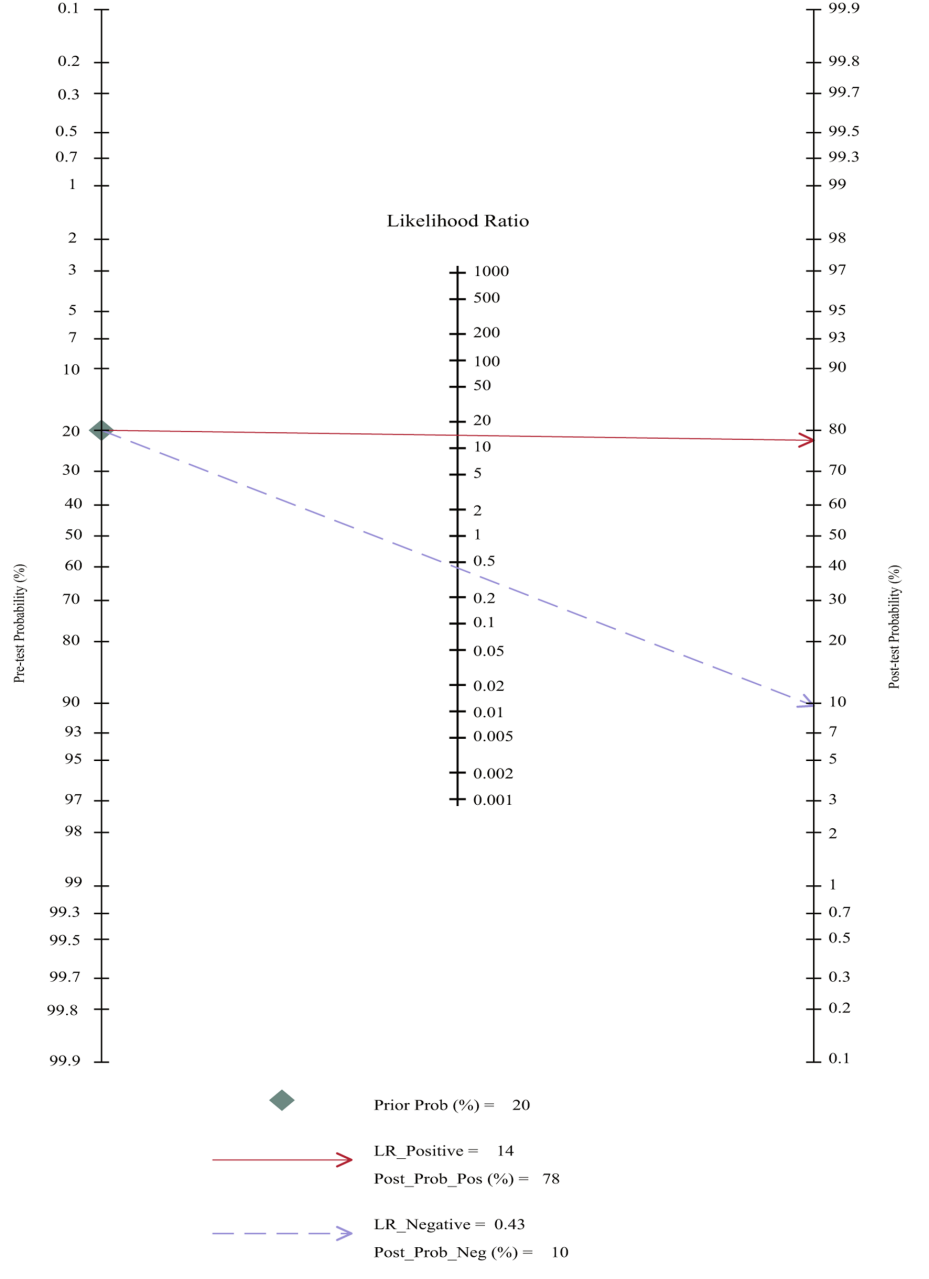
Fagan nomogram of the accuracy of BHI-V4

We evaluated the utility of BHI-V4 for diagnosing hVISA/VISA using the Fagan nomogram (Fig. 5). When the pre-test probability was set at 20%, the post-test probability was 78% for a positive result and 10% for a negative result. This indicates the good diagnostic value of BHI-V4 in clinical applications.

#### Publication bias

**Fig. 6:**
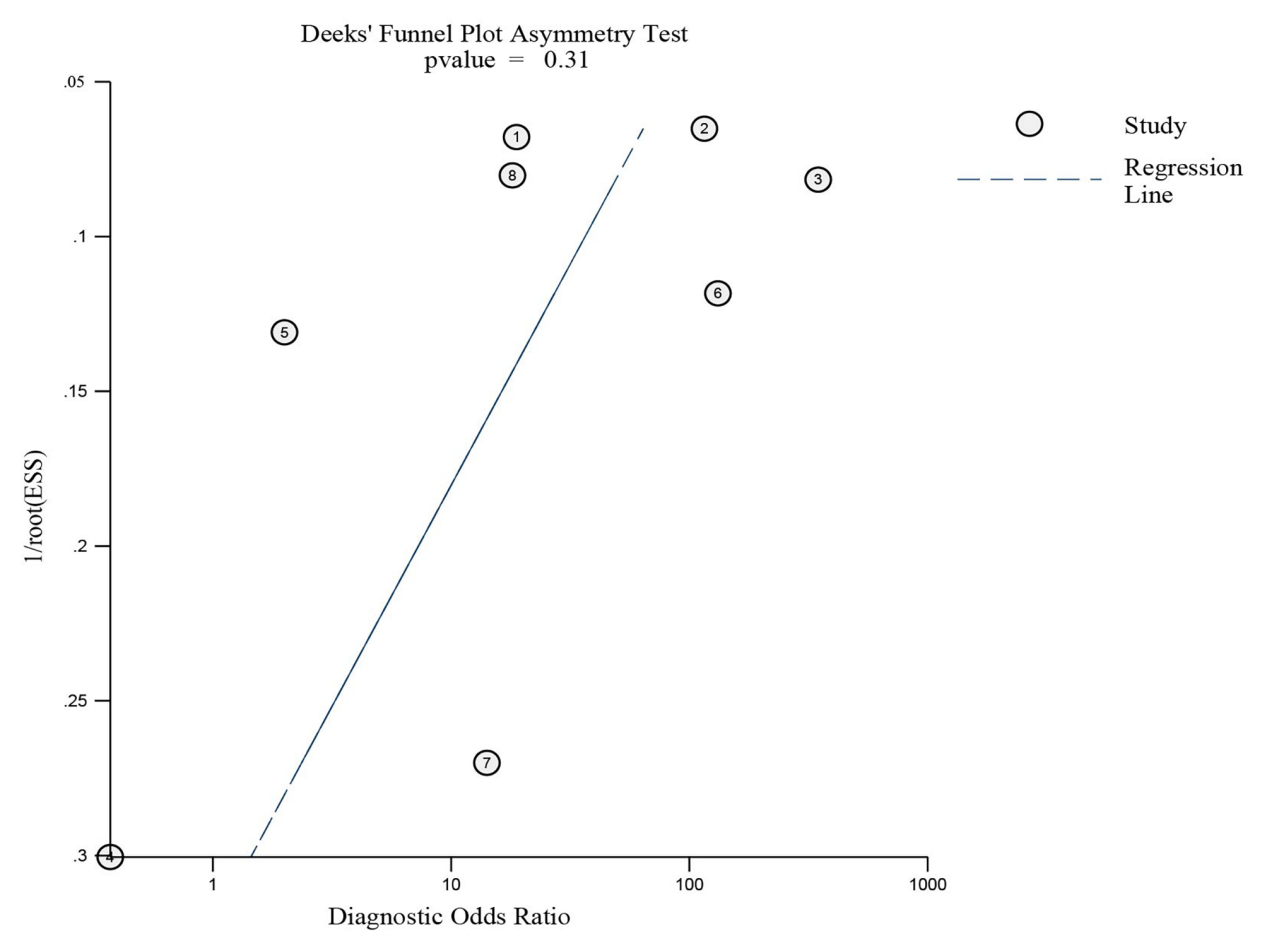
Deek funnel plot for publication bias

A Deek funnel plot (Fig. 6) was created to evaluate the presence of publication bias and there was no publication bias in the included studies for BHI-V4 in detecting hVISA/VISA (*P* = 0.31).

## Discussion

The association between the presence of hVISA/VISA and suboptimal therapeutic outcomes with glycopeptide drugs has been extensively documented in the literature. However, conventional detection techniques often face challenges in accurately identifying these bacteria due to limitations in sensitivity, potentially leading to an underestimation of their actual prevalence [20]. A meta-analysis showed that the hVISA detection rate in the 99,042 MRSA strain was 6.05%, and the VISA detection rate in the 68,792 MRSA strain was 3.01% [21]. However, the prevalence of hVISA and VISA varies greatly in different literature reports. In routine practice, it is not feasible to employ gold-standard testing for all strains. Therefore, there is an urgent need to develop a simple and cost-effective screening method that can aid in early detection and prevent antibiotic overuse. Fortunately, BHI-V4 possesses all the aforementioned advantages. While the BHI-V4 method is commonly used as a screening tool, it is important to note that its reported sensitivity and specificity have varied across different research studies [12–19], and most studies lack statistical information. Hence, our research focuses on evaluating the diagnostic value of BHI-V4 for hVISA/VISA. To the best of our knowledge, this is the first review and meta-analysis on the diagnostic method for hVISA/VISA.

This study selected eight relevant works of literature from 1232 articles using PAP-AUC as the gold standard for screening hVISA/VISA with BHI-V4. The results showed a combined sensitivity of 0.59, indicating moderate sensitivity and suggesting a potential for missed diagnoses. The specificity was 0.96, indicating strong specificity. The positive likelihood ratio was 14.0, suggesting a low misdiagnosis rate, meaning that when BHI-V4 is positive, there is a high probability of suspected hVISA/VISA. The negative likelihood ratio was 0.43, indicating a relatively high rate of missed diagnosis with BHI-V4 negativity, meaning that the possibility of excluding hVISA is relatively low when BHI-V4 is negative. The SROC AUC was 0.77, indicating high diagnostic accuracy. By comparison, BHIA supplemented with 6 µg/mL of vancomycin (BHI-V6) had a specificity of 98.8% but a sensitivity of only 3.8% while BHIA supplemented with 5 µg/mL of teicoplanin (BHI-T5) has a sensitivity of 88.5% and a specificity of 17.3% [17]. Furthermore, Etest derivative techniques such as glycopeptide resistance detection and macro Etest method have been reported to exhibit a high level of specificity but a poor level of sensitivity [5, 17, 22, 23].

The heterogeneity test results indicate that there are statistically significant differences between different research findings, which may be related to the following factors. First, BHI-V4 itself has limitations and cannot effectively distinguish between hVISA and VISA. Some studies refer to hVISA as growth on medium after 24 h, and VISA as growth on medium after 24–48 h [3, 19, 24]. However, this rough identification result may differ from the gold standard identification result. It is well-known that VISA has a more pronounced phenotype that is easier to identify than hVISA. Hence, the proportion of hVISA /VISA will affect the detection efficiency. Second, both hVISA and VISA are unstable and can revert to a sensitive state under certain conditions [25]. Therefore, timely detection is crucial for hVISA/VISA. Unfortunately, the timeliness of detection was not mentioned in the studies. Third, sample sizes vary across studies, and small sample sizes can introduce biases into the results. Four articles had a strain count of fewer than 200 cases, and three studies even had a strain count below 100 cases. In addition, various factors during the experimental process, such as the quality of the testing materials like BHI and vancomycin, incubation conditions, the status of the strains, and the proficiency of the operators, can influence the detection rate. It is difficult to identify the causes of these heterogeneity results through subgroup analysis.

There are several limitations to this article. The main limitation of this meta-analysis is that only 8 studies were included, and the heterogeneity is high. Second, because the included papers are all published, publication bias must be considered, especially in meta-analysis research that depends on published studies of testable interventions [26]. Third, the diagnostic data for BHI-V4 in the included studies were estimated indirectly, and additional direct evidence is needed to determine BHI-V4’s diagnostic significance. However, because no publication bias was identified in this investigation, we assume that this bias should be minimal.

In conclusion, our study showed that BHI-V4 had moderate diagnostic accuracy for diagnosing hVISA/VISA based on the available data. Moreover, the advantages of excellent economic benefits and simple operation make this method suitable for clinical promotion. However, its low sensitivity increases the risk of false negatives or missed detections. In clinical practice, it is reasonable to combine other screening methods such as BHI-T5 to reduce the risk of missed detections. Furthermore, more high-quality studies are needed to assess the clinical utility of BHI-V4.

### Electronic supplementary material

Below is the link to the electronic supplementary material.

Supplementary Material 1

Supplementary Material 2

## Data Availability

No datasets were generated or analysed during the current study.

## Abbreviations

BHI-V4: Brain-heart infusion agar supplemented with 4 µg/mL of vancomycin
hVISA: heteroresistant vancomycin-intermediate *Staphylococcus aureus*
VISA: vancomycin-intermediate *Staphylococcus aureus*
PAP-AUC: population analysis profiling with area under the curve
MRSA: multidrug-resistant *Staphylococcus aureus*
VRSA: vancomycin-resistant *Staphylococcus aureus*
BMD: broth microdilution method
AD: agar dilution method
TP: true positive
FP: false positive
TN: true negative
DOR: diagnostic odds ratio
SROC: summary receiver operating characteristic
BHI-V6: BHIA supplemented with 6 µg/mL of vancomycin
BHI-T5: BHIA supplemented with 5 µg/mL of teicoplanin

## References

[1] Ikuta KS, Swetschinski LR, Robles Aguilar G, Sharara F, Mestrovic T, Gray AP et al. Global mortality associated with 33 bacterial pathogens in 2019: a systematic analysis for the global burden of Disease Study 2019. Lancet. 2022;:S0140673622021857. 10.1016/S0140-6736(22)02185-7 PMC9763654 36423648

[2] Cheng X, Ma J, Su J (2022). An overview of Analytical methodologies for determination of Vancomycin in Human plasma. Molecules.

[3] Keiichi H, Aritaka N, Hanaki H, Kawasaki S, Hosoda Y, Hori S (1997). Dissemination in Japanese hospitals of strains of Staphylococcus aureus heterogeneously resistant to Vancomycin. Lancet.

[4] Maor Y, Hagin M, Belausov N, Keller N, Ben-David D, Rahav G (2009). Clinical features of Heteroresistant Vancomycin-Intermediate *Staphylococcus aureus* Bacteremia versus those of Methicillin-Resistant *S. Aureus* Bacteremia. J Infect Dis.

[5] Adam HJ, Louie L, Watt C, Gravel D, Bryce E, Loeb M (2010). Detection and characterization of heterogeneous vancomycin-intermediate *Staphylococcus aureus* isolates in Canada: results from the Canadian Nosocomial Infection Surveillance Program, 1995–2006. Antimicrob Agents Chemother.

[6] Van Hal SJ, Barbagiannakos T, Jones M, Wehrhahn MC, Mercer J, Chen D (2011). Methicillin-resistant Staphylococcus aureus Vancomycin susceptibility testing: methodology correlations, temporal trends and clonal patterns. J Antimicrob Chemother.

[7] Swenson JM, Anderson KF, Lonsway DR, Thompson A, McAllister SK, Limbago BM (2009). Accuracy of Commercial and Reference Susceptibility Testing methods for Detecting Vancomycin-Intermediate *Staphylococcus aureus*. J Clin Microbiol.

[8] Wootton M, Howe RA, Hillman R, Walsh TR, Bennett PM, MacGowan AP. A modified population analysis profile (PAP) method to detect hetero-resistance to Vancomycin in Staphylococcus aureus in a UK hospital. 2001;47:399–403. 10.1093/jac/47.4.399 11266410

[9] Page MJ, McKenzie JE, Bossuyt PM, Boutron I, Hoffmann TC, Mulrow CD et al. The PRISMA 2020 statement: an updated guideline for reporting systematic reviews. BMJ. 2021;:n71. 10.1136/bmj.n71 PMC8005924 33782057

[10] Cheng X, Zhou J, Yuan F, Ma J, Guo S, Su J. Diagnostic value of BHI-V4 for Heterogeneous and Vancomycin-Intermediate Staphylococcus aureus isolates. INPLASY. 2023. 10.1186/s12879-024-09274-4 PMC11094978 38745289

[11] Whiting PF (2011). QUADAS-2: a revised Tool for the Quality Assessment of Diagnostic Accuracy studies. Ann Intern Med.

[12] Amberpet R, Sistla S, Sugumar M, Nagasundaram N, Manoharan M, Parija S (2019). Detection of heterogeneous Vancomycin-intermediate Staphylococcus aureus: a preliminary report from south India. Indian J Med Res.

[13] Khatib R, Riederer K, Sharma M, Shemes S, Iyer SP, Szpunar S (2015). Screening for Intermediately Vancomycin-Susceptible and Vancomycin-Heteroresistant Staphylococcus aureus by Use of Vancomycin-supplemented brain heart infusion Agar biplates: defining Growth Interpretation Criteria based on gold standard confirmation. J Clin Microbiol.

[14] Riederer K, Shemes S, Chase P, Musta A, Mar A, Khatib R (2011). Detection of Intermediately Vancomycin-Susceptible and Heterogeneous Staphylococcus aureus isolates: comparison of Etest and Agar Screening methods. J Clin Microbiol.

[15] Di Gregorio S, Perazzi B, Ordoñez AM, De Gregorio S, Foccoli M, Lasala MB (2015). Clinical, microbiological, and genetic characteristics of Heteroresistant Vancomycin-Intermediate *Staphylococcus aureus* Bacteremia in a Teaching Hospital. Microb Drug Resist.

[16] Richter SS, Diekema DJ, Heilmann KP, Dohrn CL, Crispell EK, Riahi F (2014). Activities of Vancomycin, Ceftaroline, and Mupirocin against Staphylococcus aureus isolates collected in a 2011 National Surveillance Study in the United States. Antimicrob Agents Chemother.

[17] Satola SW, Farley MM, Anderson KF, Patel JB (2011). Comparison of detection methods for Heteroresistant Vancomycin-Intermediate *Staphylococcus aureus*, with the Population Analysis Profile Method as the reference method. J Clin Microbiol.

[18] da Costa TM, Morgado PGM, Cavalcante FS, Damasco AP, Nouér SA, dos Santos KRN (2016). Clinical and Microbiological Characteristics of Heteroresistant and Vancomycin-Intermediate Staphylococcus aureus from Bloodstream infections in a Brazilian Teaching Hospital. PLoS ONE.

[19] Walsh TR, Bolmström A, Qwärnström A, Ho P, Wootton M, Howe RA (2001). Evaluation of current methods for detection of Staphylococci with reduced susceptibility to Glycopeptides. J Clin Microbiol.

[20] Howden BP (2005). Recognition and management of infections caused by Vancomycin-intermediate Staphylococcus aureus (VISA) and heterogenous VISA (hVISA). Intern Med J.

[21] Zhang S, Sun X, Chang W, Dai Y, Ma X (2015). Systematic review and Meta-analysis of the epidemiology of Vancomycin-Intermediate and Heterogeneous Vancomycin-Intermediate Staphylococcus aureus isolates. PLoS ONE.

[22] Leonard SN, Rossi KL, Newton KL, Rybak MJ (2009). Evaluation of the Etest GRD for the detection of Staphylococcus aureus with reduced susceptibility to glycopeptides. J Antimicrob Chemother.

[23] Sun W, Chen H, Liu Y, Zhao C, Nichols WW, Chen M et al. Prevalence and characterization of heterogeneous vancomycin-intermediate Staphylococcus aureus isolates from 14 cities in China. ANTIMICROB AGENTS CHEMOTHER. 2009;53. 10.1128/AAC.00206-09 PMC2737858 19546358

[24] Singh A, Prasad KN, Misra R, Rahman M, Singh SK, Rai RP (2015). Increasing Trend of Heterogeneous Vancomycin Intermediate *Staphylococcus aureus* in a Tertiary Care Center of Northern India. Microb Drug Resist.

[25] Cheng X, Wang Y, Ma J, Ma L, Sun W, Su J (2023). Resistance phenotype and genetic features of a heterogeneous Vancomycin intermediate–resistant Staphylococcus aureus strain from an immunocompromised patient. Braz J Microbiol.

[26] Tacconelli E, De Angelis G, de Waure C, Cataldo MA, Torre GL, Cauda R (2009). Rapid screening tests for meticillin-resistant Staphylococcus aureus at hospital admission: systematic review and meta-analysis. Lancet Infect Dis.

