# Supplementary material for: Diagnostic value of BHI-V4 for heterogeneous and vancomycin-intermediate Staphylococcus aureus isolates: a systematic review and meta-analysis
Source: BMC Infect Dis. 2024 May 14;24:494. doi: 10.1186/s12879-024-09274-4 (PMC11094978; doi:10.1186/s12879-024-09274-4)
Supplement: Supplementary file 1 — Supplementary Material 1 [file 12879_2024_9274_MOESM1_ESM.pdf]

# Characteristics of studies

## Characteristics of included studies

### *Rajesh2017*

#### Patient Selection

| **A. Risk of Bias** | |
|---|---|
| Patient Sampling | |
| Was a consecutive or random sample of patients enrolled? | Yes |
| Was a case-control design avoided? | No |
| Did the study avoid inappropriate exclusions? | Yes |
| **Could the selection of patients have introduced bias?** | Low risk |

| **B. Concerns regarding applicability** | |
|---|---|
| Patient characteristics and setting | |
| **Are there concerns that the included patients and setting do not match the review question?** | Low concern |

#### Index Test

| | |
|---|---|
| Index tests | |

#### All tests

| **A. Risk of Bias** | |
|---|---|
| Were the index test results interpreted without knowledge of the results of the reference standard? | Yes |
| If a threshold was used, was it pre-specified? | Yes |
| **Could the conduct or interpretation of the index test have introduced bias?** | Low risk |

| **B. Concerns regarding applicability** | |
|---|---|
| **Are there concerns that the index test, its conduct, or interpretation differ from the review question?** | Low concern |

#### Reference Standard

| **A. Risk of Bias** | |
|---|---|
| Target condition and reference standard(s) | |
| Is the reference standards likely to correctly classify the target condition? | Yes |
| Were the reference standard results interpreted without knowledge of the results of the index tests? | Yes |
| **Could the reference standard, its conduct, or its interpretation have introduced bias?** | Low risk |

| **B. Concerns regarding applicability** | |
|---|---|
| **Are there concerns that the target condition as defined by the reference standard does not match the question?** | Low concern |

## Flow and Timing

| **A. Risk of Bias** | |
|---|---|
| Flow and timing | |
| Was there an appropriate interval between index test and reference standard? | Unclear |
| Did all patients receive the same reference standard? | Yes |
| Were all patients included in the analysis? | Yes |
| **Could the patient flow have introduced bias?** | Low risk |

## Notes

| **Notes** | |
|---|---|

# *Riad2015*

## Patient Selection

| **A. Risk of Bias** | |
|---|---|
| Patient Sampling | |
| Was a consecutive or random sample of patients enrolled? | Yes |
| Was a case-control design avoided? | No |
| Did the study avoid inappropriate exclusions? | Yes |
| **Could the selection of patients have introduced bias?** | Low risk |

| **B. Concerns regarding applicability** | |
|---|---|
| Patient characteristics and setting | |
| **Are there concerns that the included patients and setting do not match the review question?** | Low concern |

## Index Test

| | |
|---|---|
| Index tests | |

## All tests

| **A. Risk of Bias** | |
|---|---|
| Were the index test results interpreted without knowledge of the results of the reference standard? | Yes |
| If a threshold was used, was it pre-specified? | Yes |
| **Could the conduct or interpretation of the index test have introduced bias?** | Low risk |

| **B. Concerns regarding applicability** | |
|---|---|
| **Are there concerns that the index test, its conduct, or interpretation differ from the review question?** | Low concern |

## Reference Standard

| **A. Risk of Bias** | |
|---|---|
| Target condition and reference standard(s) | |
| Is the reference standards likely to correctly classify the target condition? | Yes |
| Were the reference standard results interpreted without knowledge of the results of the index tests? | Yes |
| **Could the reference standard, its conduct, or its interpretation have introduced bias?** | Low risk |

| **B. Concerns regarding applicability** | |
|---|---|
| **Are there concerns that the target condition as defined by the reference standard does not match the question?** | Low concern |

## Flow and Timing

| **A. Risk of Bias** | |
|---|---|
| Flow and timing | |
| Was there an appropriate interval between index test and reference standard? | Unclear |
| Did all patients receive the same reference standard? | Yes |
| Were all patients included in the analysis? | Yes |
| **Could the patient flow have introduced bias?** | Low risk |

## Notes

| | |
|---|---|
| **Notes** | |

### *Riederer2011*

#### Patient Selection

| | |
|---|---|
| **A. Risk of Bias** | |
| Patient Sampling | |
| Was a consecutive or random sample of patients enrolled? | Unclear |
| Was a case-control design avoided? | No |
| Did the study avoid inappropriate exclusions? | Unclear |
| **Could the selection of patients have introduced bias?** | Unclear risk |

| | |
|---|---|
| **B. Concerns regarding applicability** | |
| Patient characteristics and setting | |
| **Are there concerns that the included patients and setting do not match the review question?** | Low concern |

#### Index Test

| | |
|---|---|
| Index tests | |

#### All tests

| | |
|---|---|
| **A. Risk of Bias** | |
| Were the index test results interpreted without knowledge of the results of the reference standard? | Yes |
| If a threshold was used, was it pre-specified? | Yes |
| **Could the conduct or interpretation of the index test have introduced bias?** | Low risk |

| | |
|---|---|
| **B. Concerns regarding applicability** | |
| **Are there concerns that the index test, its conduct, or interpretation differ from the review question?** | Low concern |

#### Reference Standard

| **A. Risk of Bias** | |
|---|---|
| Target condition and reference standard(s) | |
| Is the reference standards likely to correctly classify the target condition? | Yes |
| Were the reference standard results interpreted without knowledge of the results of the index tests? | Yes |
| **Could the reference standard, its conduct, or its interpretation have introduced bias?** | Low risk |

| **B. Concerns regarding applicability** | |
|---|---|
| **Are there concerns that the target condition as defined by the reference standard does not match the question?** | Low concern |

## Flow and Timing

| **A. Risk of Bias** | |
|---|---|
| Flow and timing | |
| Was there an appropriate interval between index test and reference standard? | Unclear |
| Did all patients receive the same reference standard? | Yes |
| Were all patients included in the analysis? | Yes |
| **Could the patient flow have introduced bias?** | Low risk |

## Notes

| **Notes** | |
|---|---|

# *Sabrina2015*

## Patient Selection

| **A. Risk of Bias** | |
|---|---|
| Patient Sampling | |
| Was a consecutive or random sample of patients enrolled? | Yes |
| Was a case-control design avoided? | No |
| Did the study avoid inappropriate exclusions? | No |
| **Could the selection of patients have introduced bias?** | Unclear risk |

| **B. Concerns regarding applicability** | |
|---|---|
| Patient characteristics and setting | |
| **Are there concerns that the included patients and setting do not match the review question?** | Low concern |

## Index Test

| | |
|---|---|
| Index tests | |

## All tests

| **A. Risk of Bias** | |
|---|---|
| Were the index test results interpreted without knowledge of the results of the reference standard? | Yes |
| If a threshold was used, was it pre-specified? | Yes |
| **Could the conduct or interpretation of the index test have introduced bias?** | Low risk |

| **B. Concerns regarding applicability** | |
|---|---|
| **Are there concerns that the index test, its conduct, or interpretation differ from the review question?** | Low concern |

## Reference Standard

| **A. Risk of Bias** | |
|---|---|
| Target condition and reference standard(s) | |
| Is the reference standards likely to correctly classify the target condition? | Yes |
| Were the reference standard results interpreted without knowledge of the results of the index tests? | Yes |
| **Could the reference standard, its conduct, or its interpretation have introduced bias?** | Low risk |

| **B. Concerns regarding applicability** | |
|---|---|
| **Are there concerns that the target condition as defined by the reference standard does not match the question?** | Low concern |

## Flow and Timing

| **A. Risk of Bias** | |
|---|---|
| Flow and timing | |
| Was there an appropriate interval between index test and reference standard? | No |
| Did all patients receive the same reference standard? | Yes |
| Were all patients included in the analysis? | No |
| **Could the patient flow have introduced bias?** | High risk |

## Notes

| | |
|---|---|
| **Notes** | |

### *Sandra2013*

#### Patient Selection

| **A. Risk of Bias** | |
|---|---|
| Patient Sampling | |
| Was a consecutive or random sample of patients enrolled? | Unclear |
| Was a case-control design avoided? | No |
| Did the study avoid inappropriate exclusions? | Unclear |
| **Could the selection of patients have introduced bias?** | Unclear risk |

| **B. Concerns regarding applicability** | |
|---|---|
| Patient characteristics and setting | |
| **Are there concerns that the included patients and setting do not match the review question?** | Low concern |

#### Index Test

| | |
|---|---|
| Index tests | |

#### All tests

| **A. Risk of Bias** | |
|---|---|
| Were the index test results interpreted without knowledge of the results of the reference standard? | Yes |
| If a threshold was used, was it pre-specified? | Yes |
| **Could the conduct or interpretation of the index test have introduced bias?** | Low risk |

| **B. Concerns regarding applicability** | |
|---|---|
| **Are there concerns that the index test, its conduct, or interpretation differ from the review question?** | Low concern |

#### Reference Standard

| **A. Risk of Bias** | |
|---|---|
| Target condition and reference standard(s) | |
| Is the reference standards likely to correctly classify the target condition? | Yes |
| Were the reference standard results interpreted without knowledge of the results of the index tests? | Yes |
| **Could the reference standard, its conduct, or its interpretation have introduced bias?** | Low risk |

| **B. Concerns regarding applicability** | |
|---|---|
| **Are there concerns that the target condition as defined by the reference standard does not match the question?** | Low concern |

## Flow and Timing

| **A. Risk of Bias** | |
|---|---|
| Flow and timing | |
| Was there an appropriate interval between index test and reference standard? | Unclear |
| Did all patients receive the same reference standard? | Yes |
| Were all patients included in the analysis? | No |
| **Could the patient flow have introduced bias?** | Unclear risk |

## Notes

| **Notes** | |
|---|---|

# *Sarah2010*

## Patient Selection

| **A. Risk of Bias** | |
|---|---|
| Patient Sampling | |
| Was a consecutive or random sample of patients enrolled? | Unclear |
| Was a case-control design avoided? | No |
| Did the study avoid inappropriate exclusions? | Unclear |
| **Could the selection of patients have introduced bias?** | Unclear risk |

| **B. Concerns regarding applicability** | |
|---|---|
| Patient characteristics and setting | |
| **Are there concerns that the included patients and setting do not match the review question?** | Low concern |

## Index Test

| | |
|---|---|
| Index tests | |

## All tests

| A. Risk of Bias | |
|---|---|
| Were the index test results interpreted without knowledge of the results of the reference standard? | Yes |
| If a threshold was used, was it pre-specified? | Yes |
| **Could the conduct or interpretation of the index test have introduced bias?** | Low risk |

| B. Concerns regarding applicability | |
|---|---|
| **Are there concerns that the index test, its conduct, or interpretation differ from the review question?** | Low concern |

## Reference Standard

| A. Risk of Bias | |
|---|---|
| Target condition and reference standard(s) | |
| Is the reference standards likely to correctly classify the target condition? | Yes |
| Were the reference standard results interpreted without knowledge of the results of the index tests? | Yes |
| **Could the reference standard, its conduct, or its interpretation have introduced bias?** | Low risk |

| B. Concerns regarding applicability | |
|---|---|
| **Are there concerns that the target condition as defined by the reference standard does not match the question?** | Low concern |

## Flow and Timing

| A. Risk of Bias | |
|---|---|
| Flow and timing | |
| Was there an appropriate interval between index test and reference standard? | Unclear |
| Did all patients receive the same reference standard? | Yes |
| Were all patients included in the analysis? | Yes |
| **Could the patient flow have introduced bias?** | Low risk |

## Notes

| | |
|---|---|
| **Notes** | |

### *Thaina2016*

#### Patient Selection

| | |
|---|---|
| **A. Risk of Bias** | |
| Patient Sampling | |
| Was a consecutive or random sample of patients enrolled? | Unclear |
| Was a case-control design avoided? | No |
| Did the study avoid inappropriate exclusions? | Unclear |
| **Could the selection of patients have introduced bias?** | Unclear risk |

| | |
|---|---|
| **B. Concerns regarding applicability** | |
| Patient characteristics and setting | |
| **Are there concerns that the included patients and setting do not match the review question?** | Low concern |

#### Index Test

| | |
|---|---|
| Index tests | |

#### All tests

| | |
|---|---|
| **A. Risk of Bias** | |
| Were the index test results interpreted without knowledge of the results of the reference standard? | Yes |
| If a threshold was used, was it pre-specified? | Yes |
| **Could the conduct or interpretation of the index test have introduced bias?** | Low risk |

| | |
|---|---|
| **B. Concerns regarding applicability** | |
| **Are there concerns that the index test, its conduct, or interpretation differ from the review question?** | Low concern |

#### Reference Standard

| **A. Risk of Bias** | |
|---|---|
| Target condition and reference standard(s) | |
| Is the reference standards likely to correctly classify the target condition? | Yes |
| Were the reference standard results interpreted without knowledge of the results of the index tests? | Yes |
| **Could the reference standard, its conduct, or its interpretation have introduced bias?** | Low risk |

| **B. Concerns regarding applicability** | |
|---|---|
| **Are there concerns that the target condition as defined by the reference standard does not match the question?** | Low concern |

## Flow and Timing

| **A. Risk of Bias** | |
|---|---|
| Flow and timing | |
| Was there an appropriate interval between index test and reference standard? | Unclear |
| Did all patients receive the same reference standard? | Yes |
| Were all patients included in the analysis? | No |
| **Could the patient flow have introduced bias?** | High risk |

## Notes

| **Notes** | |
|---|---|

# *Timothy2001*

## Patient Selection

| **A. Risk of Bias** | |
|---|---|
| Patient Sampling | |
| Was a consecutive or random sample of patients enrolled? | Unclear |
| Was a case-control design avoided? | No |
| Did the study avoid inappropriate exclusions? | Unclear |
| **Could the selection of patients have introduced bias?** | Unclear risk |

| **B. Concerns regarding applicability** | |
|---|---|
| Patient characteristics and setting | |
| **Are there concerns that the included patients and setting do not match the review question?** | Low concern |

## Index Test

| | |
|---|---|
| Index tests | |

## All tests

| **A. Risk of Bias** | |
|---|---|
| Were the index test results interpreted without knowledge of the results of the reference standard? | Yes |
| If a threshold was used, was it pre-specified? | Yes |
| **Could the conduct or interpretation of the index test have introduced bias?** | Low risk |

| **B. Concerns regarding applicability** | |
|---|---|
| **Are there concerns that the index test, its conduct, or interpretation differ from the review question?** | Low concern |

## Reference Standard

| **A. Risk of Bias** | |
|---|---|
| Target condition and reference standard(s) | |
| Is the reference standards likely to correctly classify the target condition? | Yes |
| Were the reference standard results interpreted without knowledge of the results of the index tests? | Yes |
| **Could the reference standard, its conduct, or its interpretation have introduced bias?** | Low risk |

| **B. Concerns regarding applicability** | |
|---|---|
| **Are there concerns that the target condition as defined by the reference standard does not match the question?** | Low concern |

## Flow and Timing

| **A. Risk of Bias** | |
|---|---|
| Flow and timing | |
| Was there an appropriate interval between index test and reference standard? | Unclear |
| Did all patients receive the same reference standard? | No |
| Were all patients included in the analysis? | Yes |
| **Could the patient flow have introduced bias?** | High risk |

## Notes

| | |
|---|---|
| **Notes** | |

*Footnotes*

### Characteristics of excluded studies

*Footnotes*

### Characteristics of studies awaiting classification

*Footnotes*

### Characteristics of ongoing studies

*Footnotes*

# Summary of findings tables

# Additional tables
