# Supplementary figures and images for: Diagnostic value of BHI-V4 for heterogeneous and vancomycin-intermediate Staphylococcus aureus isolates: a systematic review and meta-analysis
Source: BMC Infect Dis. 2024 May 14;24:494. doi: 10.1186/s12879-024-09274-4 (PMC11094978; doi:10.1186/s12879-024-09274-4)

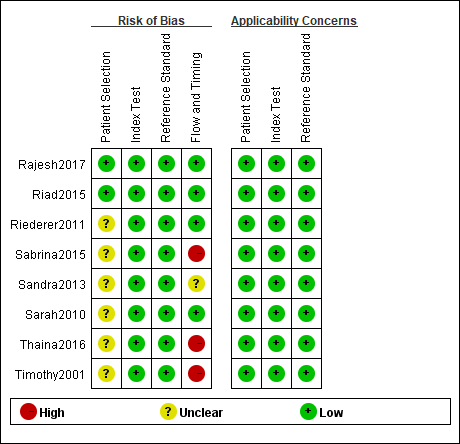

Supplement: Supplementary file 2 — Supplementary Material 2 [file 12879_2024_9274_MOESM2_ESM.png]
